# Radial Reconstruction With Fibular Free Flap Using CAD‐CAM and Virtual Surgical Planning in Pediatric Pseudoarthrosis due to Neurofibromatosis Type 1: A Case Report and Literature Review

**DOI:** 10.1002/micr.70285

**Published:** 2026-08-27

**Authors:** Alicia Dean, Manuel Román Torres, Concepción Centella Gutiérrez, Guillermo Rodríguez Martínez, Genoveva Molina Pérez, Francisco Alamillos Granados

**Affiliations:** ^1^ Department of Oral and Maxillofacial Surgery Hospital Universitario Reina Sofía Córdoba Spain; ^2^ Department of Orthopedic Surgery and Traumatology Hospital Universitario Reina Sofía Córdoba Spain

**Keywords:** CAD‐CAM design, congenital pseudoarthrosis, fibular free flap, neurofibromatosis type 1, pediatric forearm reconstruction, virtual surgical planning

## Abstract

Neurofibromatosis type 1 (NF1) is an autosomal dominant disorder that causes skeletal dysplasia and congenital pseudoarthrosis of the forearm, which is very difficult to repair. The vascularized free fibular flap (FFF) is the standard treatment. CAD‐CAM technologies and virtual surgical planning (VSP) offer greater precision and their full integration into primary pediatric reconstruction for NF1 is described. The objective of this case report is to present the simultaneous integration of all these tools in the primary reconstruction of a child with forearm pseudarthrosis due to NF1. We present the case of a 7‐year‐old boy with NF1 and radial pseudoarthrosis reconstructed using FFF, patient‐specific CAD‐CAM guides, and an interactive 3D VSP environment. A 3‐cm defect was reconstructed using an FFF and a premolded LCP plate. Bone union was achieved at 7–8 months, with full range of motion in the wrist and nearly complete pronation‐supination. No morbidity was reported in the donor site. This case reports the complete integration of FFF, CAD‐CAM, and VSP in the primary reconstruction of a forearm pseudarthrosis in a child with NF1.

**Level of Evidence:**

V Expert Opinion/Case Report.

## 1 Introduction

Neurofibromatosis type 1 (NF1) is an autosomal dominant hereditary disorder in which skeletal dysplasia can lead to anterolateral deformity and congenital pseudoarthrosis of the long bones (Jayakrishnan and Sebastian 2020; Katchburian et al. 2024). Although the tibia is the most commonly affected bone, involvement of the forearm is well documented, and once pseudarthrosis develops, it is very difficult to achieve healing (Katchburian et al. 2024; Grünberger et al. 2023). Approximately 5% of patients with NF1 develop pseudarthrosis, and about half of all cases of congenital pseudarthrosis are associated with NF1; just over a hundred cases have been reported in the forearm (Katchburian et al. 2024).

Free fibular flap (FFF) has become the standard treatment for diaphyseal reconstruction, as it provides length and cortical strength with adequate blood supply (Grünberger et al. 2023; Siebelt et al. 2020; Witoonchart et al. 1999; Adani et al. 2004; Malizos et al. 2004). Failures are often related to incomplete resection of the dysplastic bone and insufficient stability.

CAD‐CAM and virtual surgical planning (VSP) improve precision through three‐dimensional (3D) segmentation, simulated osteotomies, patient‐specific cutting guides, and premolding of the plate on a stereolithographic model of the reconstructed bone (Ettinger et al. 2018; Alperovich et al. 2017; Pereira et al. 2022; Zaussinger et al. 2025). Despite their advantages, reports on the forearm describe either conventional approaches without digital planning (Ding et al. 2017) or digital planning applied solely to secondary corrective procedures (Grünberger et al. 2023; Hansen and Sethi 2025). The objective of this case report is to present the first simultaneous integration of FFF, CAD‐CAM, and VSP in the primary reconstruction of a child with forearm pseudoarthrosis associated with NF1.

## 2 Case Report

We present the case of a 7‐year‐old boy with NF1 (maternal family history) and pseudoarthrosis of the left forearm. His medical history included nonunion of the ulna (treated conservatively 3 years before) and a subsequent pathological fracture of the radius following a minor trauma. Conservative treatment failed; radiographs showed persistent atrophic pseudoarthrosis with progressive angular deformity.

**FIGURE 1:**
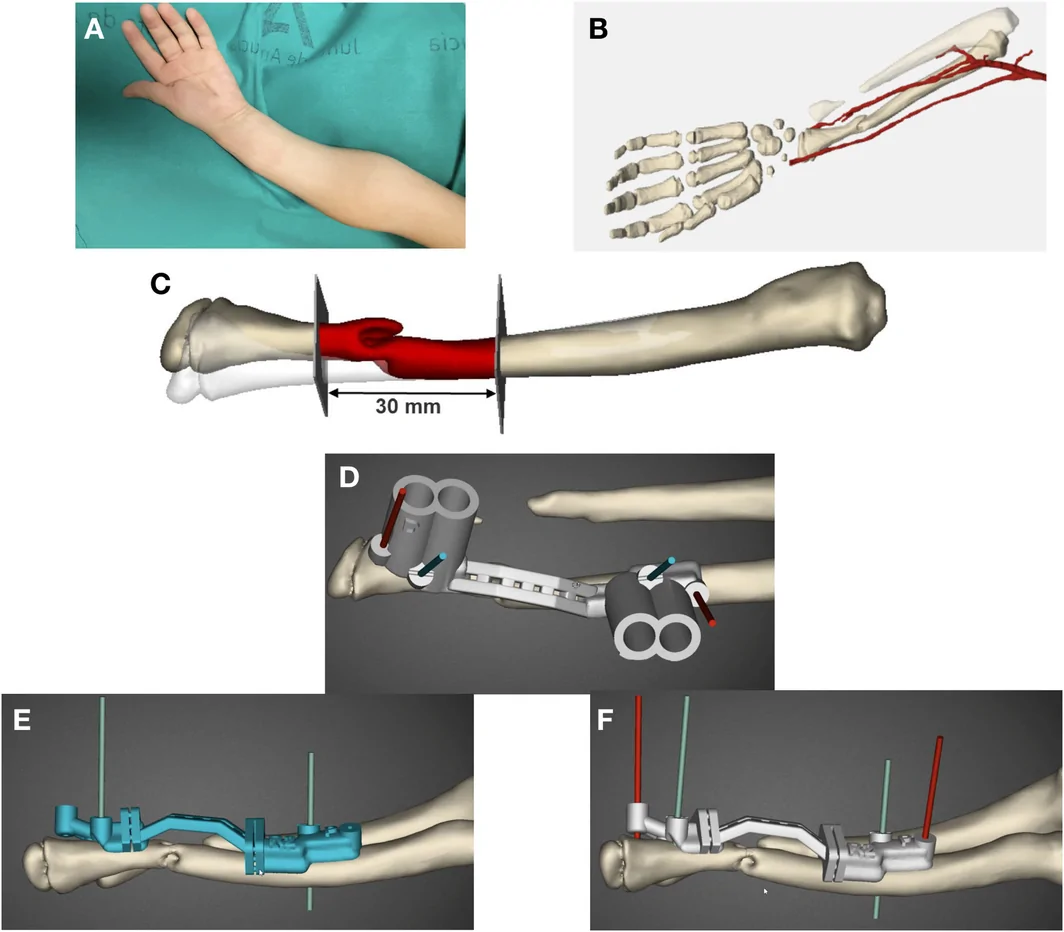
(A) Clinical aspect of the left forearm. (B) Auto segmentation of the ulna and radius and radial and ulnar arteries of the forearm. Pseudoarthrosis of the radius is noted. (C) Planned resection of the radius. (D) Surgical guide 1 (positioning guide), for drilling holes for the osteosynthesis plate. The surgical guide contains information on the necessary movements of the radius to achieve alignment according to the plan made by superimposing the mirror image of the healthy side. (E) The first surgical guide of the forearm is removed. Two of the Kirchner wires are kept in place. (F) The second surgical guide (cutting guide) is placed over the Kirchner wires that have been retained, and two new Kirchner wires are added for stability. This second guide is used to perform the planned osteotomy of the radius bone.

Preoperative radiographs, computed tomography (CT) scans, CT angiography, and magnetic resonance imaging (MRI) were obtained to determine bone involvement, evaluate the anatomy of the recipient vessels, and plan the resection. Thin‐section (≤ 1 mm) CT images of both extremities were imported into Mimics/3‐matic (Materialise NV, Leuven, Belgium) for: (1) simulation of resection down to healthy bone; (2) contralateral mirror superimposition for rotational correction; (3) virtual positioning of the FFF; and (4) placement of the LCP plate with planned screw trajectories. STL files were generated for all components (Figure 1).

**FIGURE 2:**
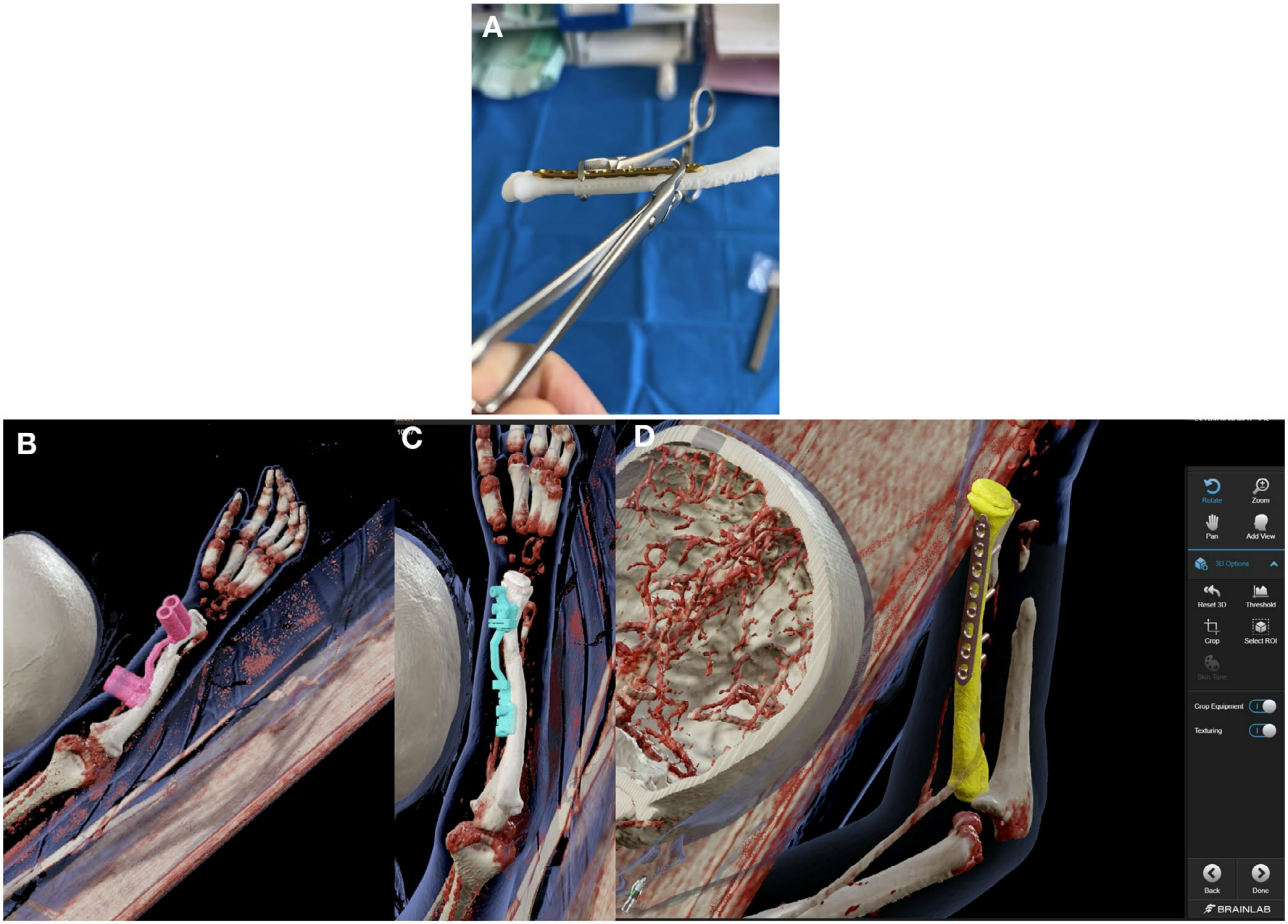
(A) The osteosynthesis plate used was molded on a stereolithographic model of the radial reconstruction with the FFF. Marking the exact position of the plate helped to make a more accurate molding of the plate. (B–D) All STL objects (bone resection, guides, free fibular flap [FFF], planned plate and screws) were imported into Brainlab Elements navigation system software for surgical reference.

The cutting and positioning guides were manufactured from PA12 nylon (Materialise NV). A 2.4‐mm, eight‐hole pediatric LCP plate (DePuy Synthes) was hand‐contoured over the stereolithographic model. The recipient vessels were evaluated using CT angiography and color Doppler ultrasound; CT angiography was indicated given the known association of NF1 with arterial dysplasia. All STL objects were imported into Brainlab Elements (Brainlab AG, Munich, Germany) as a fully interactive 3D reference environment in which the surgical team could rotate the complete surgical model and select or deselect individual components (bone, guides, flap, plate, skin). Active intraoperative navigation was not used (Figures 1 and 2).

**FIGURE 3:**
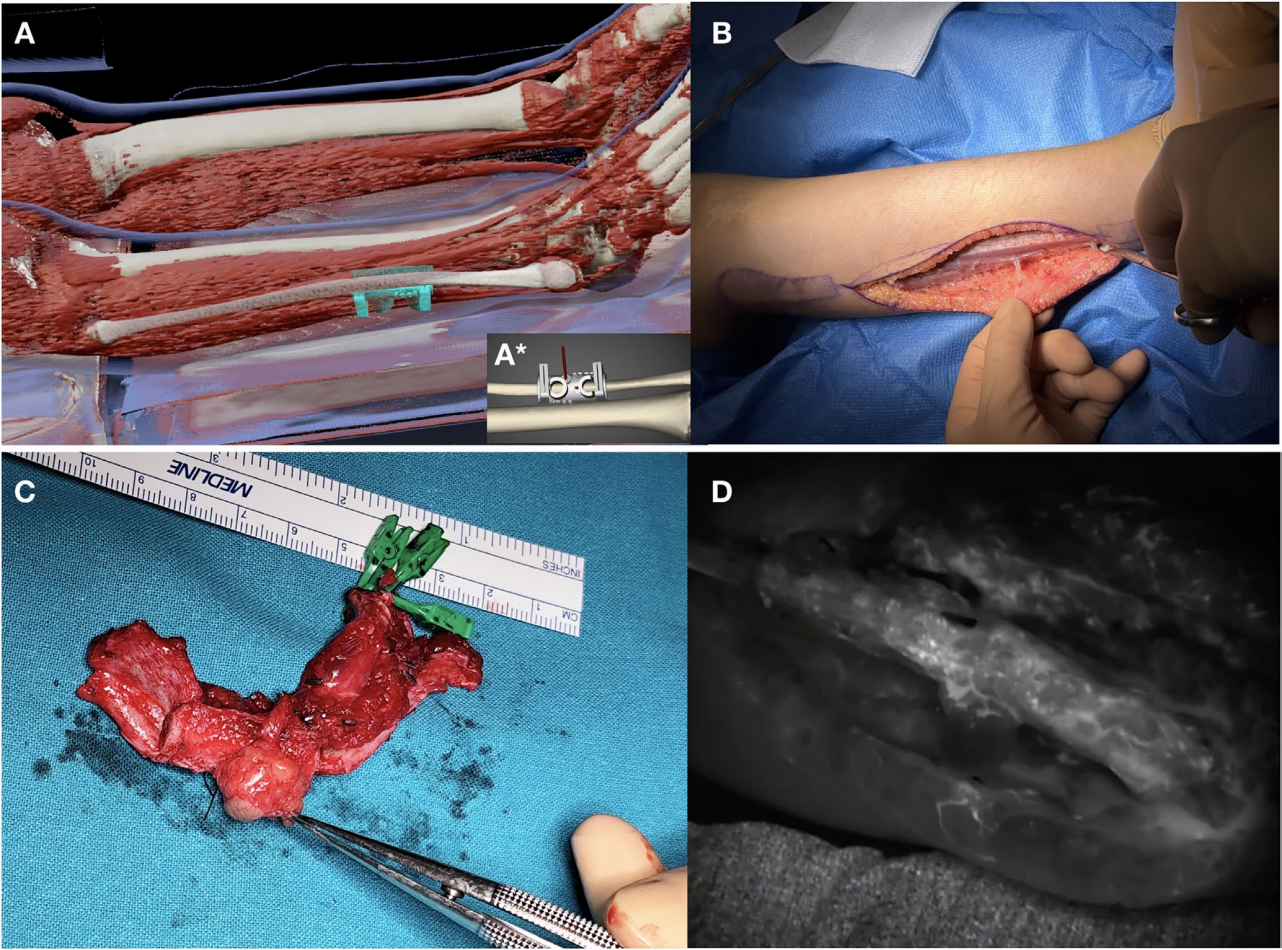
(A) Fibula surgical guide into Brain Lab's Elements navigation system software for surgical reference. (A*) A close‐up of the fibula surgical guide with holes for two screws used to attach the FFF to the osteosynthesis plate. (B) FFF with a cutaneous perforator. (C) Periosteal strip can be appreciated on both sides of the bone to circumferentially wrap the junction between the fibula and the recipient radial bone, in order to increase vascularization at the junction and improve the healing process. (D) Intraoperative fluorescence imaging (indocyanine green) demonstrating complete revascularization of the FFF.

**FIGURE 4:**
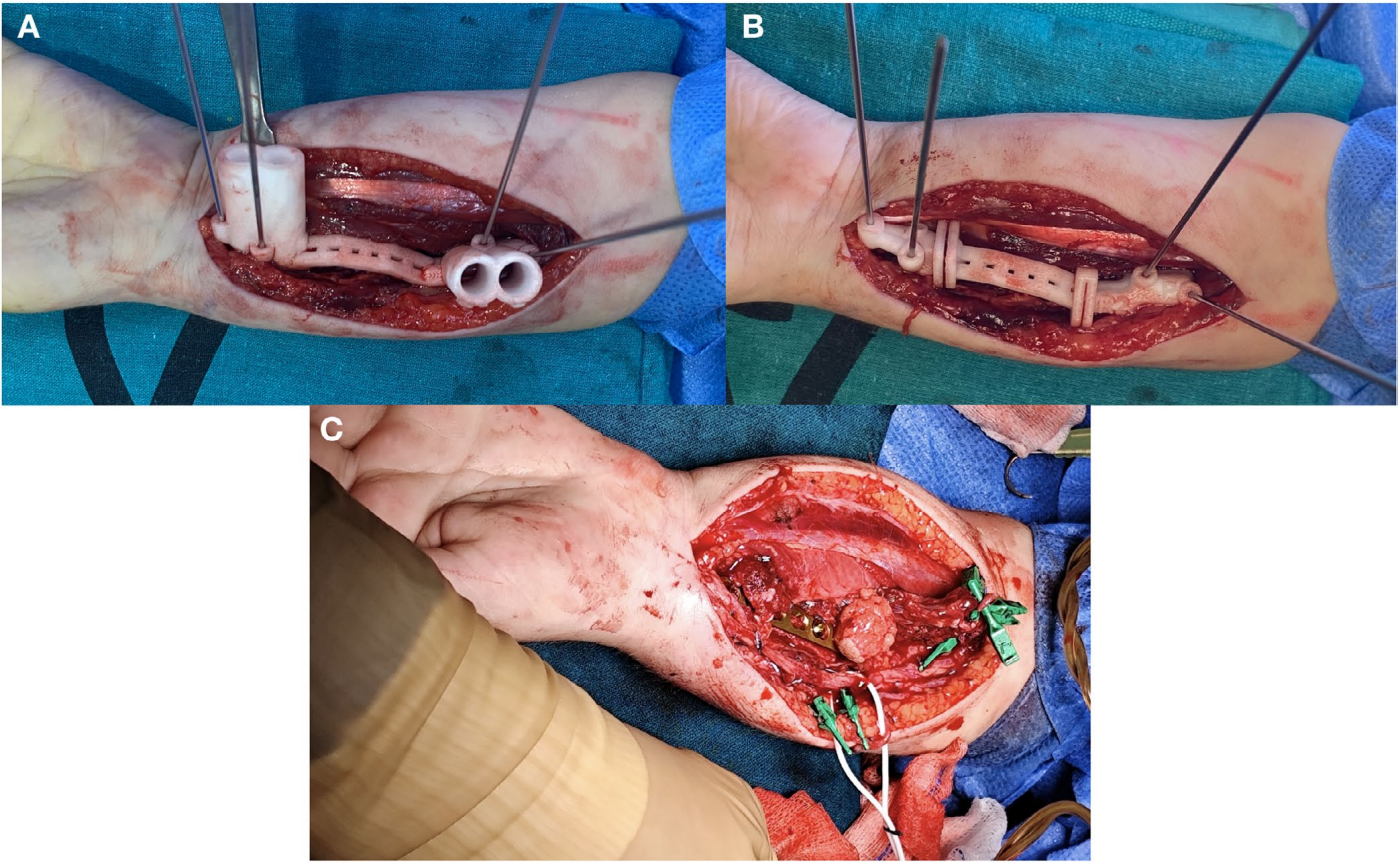
(A) First surgical guide during surgery. (B) Second surgical guide during surgery. (C) FFF in the correct position with osteosynthesis before vascular anastomosis.

Using a Henry volar approach and patient‐specific guides, the dysplastic radial segment was resected to healthy bone, and the abnormal periosteum removed, leaving a 3‐cm defect. A FFF was harvested from the right fibula with the peroneal pedicle, preserving 7 cm proximally and 7 cm distally; a monitoring skin paddle (10 × 5 mm) and periosteal strips for fibular‐radius junctions were included; distal tibiofibular arthrodesis was performed. The FFF was inserted to the planned length, alignment, and rotation, and the preformed plate applied. Distal radioulnar fixation was not possible given the absence of the distal ulnar epiphysis from prior pseudoarthrosis. Peroneal–radial end‐to‐side arterial and cephalic venous end‐to‐end anastomoses were performed; perfusion was confirmed by ICG fluorescence angiography (Fluobeam, Fluoptics, Grenoble, France) (Figures 2, 3, 4). Ethics committee approval was not required (Helsinki Declaration); written parental consent was obtained.

The postoperative course was uneventful. Serial radiographs showed cortical union by 7–8 months. The child recovered full pain‐free wrist motion: flexion 90°, extension 80°, ulnar deviation 40°, radial deviation 15°, and near‐complete pronation–supination, comparable to the contralateral side, with no donor‐site morbidity. After 4 weeks in a plaster splint, a thermoplastic orthosis (EXOS) was worn 23 h/day with assisted exercises from month 1; a ≈20° wrist‐extension deficit at 3 months resolved completely, and the orthosis was discontinued from Month 4 (Figure S1).

## 3 Discussion

Several authors have described the treatment of forearm pseudoarthrosis in NF1 with FFF (Sellers et al. 1988; Allieu et al. 1999; Mathoulin et al. 1993; Masterson et al. 1993; Cheng et al. 1994; Ramelli et al. 2001; Bae et al. 2005; El Hage et al. 2009; Beris et al. 2010; Bauer et al. 2013) (Table 1). The literature was identified through a non‐systematic search of PubMed/MEDLINE, Embase, and Web of Science using terms including “congenital pseudoarthrosis,” “neurofibromatosis type 1,” “free fibular flap,” “CAD‐CAM,” and “virtual surgical planning,” with no date restriction. FFF achieves union in 4–8 months with good functional recovery and low donor‐site morbidity (Adani et al. 2004; Malizos et al. 2004; Cano‐Luís et al. 2018). Complete resection of dysplastic bone and rigid fixation are key for success (Katchburian et al. 2024). FFF also proves effective following failed non‐vascularized reconstructions (Pamuk et al. 2023).

**TABLE 1:** Published reports relevant to forearm reconstruction with fibular free flap (FFF) in pediatric patients with NF1 and CAD‐based planning, plus the present case.

First author	Year	Number of cases	Type of article	Age (years) patients with NF	Sex (male/female)	FFF	CAD‐CAM design	VSP	Complications	Time to consolidation	Functional outcomes
Sellers	1988	1 (with NF)	Case report	4	1/0	Yes	No	No	None reported	6 weeks	Full range of motion
Bell^a^	1989	6 (1 with NF and FFF)	Case series	3	1/0	Yes (only one case)	No	No	None reported	6 months	—
Mathoulin	1993	6 (5 with NF1)	Case series	1, 3, 7, 9, 13	4/1	Yes	No	No	1 case graft fracture 5 years later	Mean 6 months	Motion was practically normal except supination which was limited in all cases. Shortening was always moderate (2 cm)
Masterson	1993	1 (with NF1)	Case report	5	0/1	Yes	No	No	None reported	9 months	Normal function
Cheng	1994	3 (2 with NF, 1 with FFF)	Case series	9 months	0/1	Yes (only one case)	No	No	None reported	8 months	Normal function
Witoonchart	1999	3 (2 with NF1)	Case series	2, 5	0/2	Yes	No	No	None reported	5–7 months	Normal function, full mobility
Allieu	1999	2 (1 with NF1)	Case series	4	0/1	Yes	No	No	Shortening	2 months	Excellent elbow and wrist function. Residual clinical shortening
Ramelli	2001	2 (both with NF1). Only one with FFF	Case series	2	0/1	Yes	No	No	None reported	6 months	Normal function, acceptable mobility
Bae	2005	4 (3 with NF1)	Case series	3, 5, 16	2/2	Yes	No	No	Symptomatic hardware	6 months	Normal function, acceptable mobility
El Hage	2009	2 (with NF)	Case series	1.5, 12	0/2	Yes	No	No	Proximal ulnar malunion. Proximal ulnofibular nonunion. Radial head dislocation	6 weeks and 3 months	Acceptable mobility
Beris	2010	1 (with NF1)	Case report	9	0/1	Yes	No	No	None reported	Union at 4 months, 10‐year follow‐up	Full union, complete wrist/forearm motion
Bauer	2013	5 (3 with NF1)	Case series	9, 2, 12	3/0	Yes	No	No	Ulna nonunion, limited pronation	Mean of 10 months (range 3–18 months)	2 patients with full range of motion. 1 patient limited pronation
Ding	2017	1 (with NF1)	Case report	7	1/0	Yes	No	No	None reported	6 months	Full union, preserved growth, good wrist/forearm motion
Siebelt	2020	84 (systematic review; majority with NF1)	Systematic review/case series	Mostly < 12	Not specified	Yes	Not reported	Not reported	13% non‐union, rare infection, occasional refracture	Median 6–12 months (FFF)	FFF showed high unión rates (87%).
Grünberger	2023	1 (with NF1)	Case report + review	12	1/0	Yes	Yes (secondary surgery no with the FFF)	No	Secondary surgery for radial bowing	3 months (primary), 6 years (correction)	Pain‐free, unrestricted function
Katchburian	2024	3 (all with NF1)	Technical report + case series	3, 9, 9	1/2	Yes	No	No	1 persistent nonunion (revised), secondary surgery common	Up to 11 months	Union achieved, good function, rigid fixation emphasized
Dean (current case)	2025	1 (all with NF1)	Technical report + review	7	1/0	Yes	Yes	Yes	No	7–8 months	Union achieved, good function, rigid fixation emphasized

*Note:* Inclusion: pseudoarthrosis forearm, NF1, pediatric, treated with FFF. Exclusion: posttraumatic and post‐oncologic defects.

Abbreviations: CAD‐CAM, computer‐aided design/manufacturing; FFF, free fibular flap; NF1, neurofibromatosis type 1; VSP, virtual surgical planning.

^a^ Referred in Grünberger et al. (2023).

The practical value of this workflow lies in several concrete contributions to surgical planning and execution that deserve analysis.

The two software platforms serve distinct and sequential roles. Materialise Mimics/3‐matic is the active planning and CAD‐CAM design environment: it performs bone segmentation, contralateral mirror superimposition, virtual osteotomy simulation, flap positioning, drill trajectory and screw depth planning, plate placement, 3D model printing for plate premolding, and design of patient‐specific cutting and positioning guides, exporting all outputs as STL files (Ettinger et al. 2018; Alperovich et al. 2017; Pereira et al. 2022; Zaussinger et al. 2025; Hansen and Sethi 2025). Brainlab Elements then functions as an advanced multimodal viewer: it imports these STL objects together with the patient's imaging data to create a fully interactive 3D reference environment. At the donor site it imports the fibular cutting guide, identifies the optimal cutaneous perforator, and visualizes the fibular flap vasculature; at the recipient forearm it calculates the optimal pedicle‐to‐recipient vessel distance. Individual components can be toggled on and off and volumetric superimposition performed. It provides spatial reference and measurement capabilities that extend meaningfully beyond static imaging.

Active navigation was not used: the CAD‐CAM guides provided sufficient precision and, furthermore, the poor bone quality and limited cortical diameter of the dysplastic radial shaft precluded reliable placement of a reference array for patient registration. Bilateral extremity CT is irreplaceable, as no alternative provides the volumetric precision needed for guide design, rotational planning, and pedicle measurement.

VSP reduces intraoperative uncertainty: when 3D bone movements are needed, rotation, realignment, and translation of the residual bone and the reconstructed segment, planning them in advance makes it easier to achieve the surgical objectives. The use of contralateral mirror symmetry enhances the goal of 3D bone reconstruction. The entire surgical team can anticipate potential complications before entering the operating room, which improves outcomes and the patient's quality of life. By reducing uncertainty, operative time is shortened, which directly translates to a shorter total duration of the procedure and lower procedural costs. The cutting guides were easy to place and use in this case, and the availability of the complete surgical plan within the Brainlab Elements environment served as a continuous intraoperative visual reference throughout the entire procedure.

Ionizing radiation exposure in children should always be carefully considered. Each imaging study in this case was individually justified by clinical necessity, but reconstructive surgeons must weigh the risk–benefit balance of every radiological study in pediatric patients, following the “as low as reasonably achievable” principle (Smith‐Bindman et al. 2025; Bosch de Basea et al. 2023).

Long‐term follow‐up is critical. The distal tibiofibular arthrodesis performed at the donor site prevents ankle instability and growth‐related deformity, and progressive remodeling of the FFF within the growing radius is expected (Bae et al. 2005; El Hage et al. 2009). As the FFF lacks intrinsic growth potential, limb length discrepancy must be monitored until skeletal maturity, and in very young children the timing of surgery must weigh the risk of progressive deformity against the potential for donor‐site growth disturbance. Follow‐up until skeletal maturity is planned.

Single‐case evidence (Level V), nonsystematic review, and limited follow‐up are the main limitations of this work. Future studies should address efficiency gains, growth remodeling, and preformed versus customized plates (Alperovich et al. 2017).

We describe a step‐by‐step workflow combining patient‐specific CAD‐CAM guides, an interactive 3D VSP environment, and FFF in the primary reconstruction of pediatric NF1 forearm pseudoarthrosis. The protocol, contralateral mirror planning, patient‐specific guides, model‐based plate premolding, interactive 3D visualization, and ICG monitoring is reproducible and transferable. Future studies should address efficiency gains, growth outcomes, and optimal plate strategies (Ettinger et al. 2018; Alperovich et al. 2017; Pereira et al. 2022; Zaussinger et al. 2025).

## Author Contributions

**Alicia Dean:** conceptualization, methodology, surgical planning, surgery (lead surgeon), writing (original draft, writing) review and editing, supervision. **Manuel Román Torres:** surgery (orthopedic co‐surgeon), data collection, review and editing. **Concepción Centella Gutiérrez:** surgery (microsurgery), data collection, review and editing. **Guillermo Rodríguez Martínez:** surgery (orthopedic), data collection, review and editing. **Genoveva Molina Pérez:** surgery (microsurgery), data collection, review and editing. **Francisco Alamillos Granados:** virtual surgical planning, CAD‐CAM design, surgery, supervision, review and editing.

## Funding

The authors have nothing to report.

## Ethics Statement

Formal ethics committee approval was not required for this single anonymous case report, as it does not meet the criteria for studies requiring institutional review board oversight under applicable national regulations. The work was carried out in accordance with the Declaration of Helsinki.

## Consent

Written informed consent was obtained from the patient's parents/guardians for the publication of clinical details and images, including radiographs, three‐dimensional reconstructions, and intraoperative indocyanine green fluorescence images. All patient identifiers were removed and images were cropped to prevent recognition. The original signed consent form is held by the treating institution (Hospital Universitario Reina Sofía, Córdoba, Spain).

## Conflicts of Interest

Dr. Alicia Dean has served as a speaker at webinars organized by Brainlab AG (Munich, Germany), the manufacturer of the Brainlab Elements software used in this case. The other authors declare no conflicts of interest.

## Supporting information


**Figure S1:** (A) Postoperative clinical view of the forearm. (B) Postoperative X‐ray. (C) Clinical view of the donor leg of the FFF.

## Data Availability

The data that support the findings of this study are available on request from the corresponding author. The data are not publicly available due to privacy or ethical restrictions.
